# Effect of Nanoporous Molecular Sieves TS-1 on Electrical Properties of Crosslinked Polyethylene Nanocomposites

**DOI:** 10.3390/nano14110985

**Published:** 2024-06-06

**Authors:** Lirui Shi, Chong Zhang, Zhaoliang Xing, Yuanyi Kang, Weihua Han, Meng Xin, Chuncheng Hao

**Affiliations:** 1 State Key Laboratory of Advanced Power Transmission Technology, Beijing 102209, China; shilirui0302@163.com (L.S.); zhangc@sgcc.com.cn (C.Z.); xingzhaoliang007@163.com (Z.X.); 2 College of Materials Science and Engineering, Qingdao University of Science and Technology, Qingdao 266042, China; kangyuanyi1080@163.com (Y.K.); vienhan@163.com (W.H.); xinmeng_7591@126.com (M.X.)

**Keywords:** crosslinked polyethylene composites, aperture structure, thermal conductivity, space charge, breakdown field strength

## Abstract

Crosslinked polyethylene (XLPE) is an important polyethylene modification material which is widely used in high-voltage direct current (HVDC) transmission systems. Low-density polyethylene (LDPE) was used as a matrix to improve the thermal and electrical properties of XLPE composites through the synergistic effect of a crosslinking agent and nanopore structure molecular sieve, TS-1. It was found that the electrical and thermal properties of the matrices were different due to the crosslinking degree and crosslinking efficiency, and the introduction of TS-1 enhanced the dielectric constants of the two matrices to 2.53 and 2.54, and the direct current (DC) resistivities were increased to 3 × 10^12^ and 4 × 10^12^ Ω·m, with the enhancement of the thermal conductivity at different temperatures. As the applied voltage increases, the DC breakdown field strength is enhanced from 318 to 363 kV/mm and 330 to 356 kV/mm. The unique nanopore structure of TS-1 itself can inhibit the injection and accumulation in the internal space of crosslinked polyethylene composites, and the pore size effect of the filler can limit the development of electron impact ionization, inhibit the electron avalanche breakdown, and improve the strength of the external applied electric field (breakdown field) that TS-1/XLPE nanocomposites can withstand. This provides a new method for the preparation of nanocomposite insulating dielectric materials for HVDC transmission systems with better performance.

## 1. Introduction

High-voltage direct current (HVDC) transmission cables play an important role in the HVDC transmission system, and their safe operation is related to the stability of the whole system [1]. Crosslinked polyethylene, as a key insulating material for extruded HVDC transmission cables, has been widely used for its excellent electrical insulating properties, very high electrical resistivity, reliability, availability, and low cost [2]. In practice, it has been found that under the prolonged action of a high-voltage direct current electric field, the insulating properties of the material gradually degrade under the combined stress of thermal, electrical, and chemical factors [3,4]. The accumulation of space charge inside the material can cause serious distortion of the local electric field, resulting in localized heating and localized discharges [5,6], and in the worst case, it may lead to insulation breakdown [7,8]. This can reduce the strength of the insulation and create a potential safety hazard. In addition, space charge accumulation and thermal aging not only affect the physical and chemical properties of electrical insulating materials but also lead to other degradation phenomena. Therefore, suppressing space charge accumulation and improving the insulation strength and thermodynamic properties of insulating materials are of great significance for the further development of DC power transmission technology.

XLPE is made of modified polyethylene, whose polymer chains are chemically crosslinked with excellent chemical stability, electrical properties, thermomechanical stability, and processability [2,9]. In addition, a large number of studies have shown that the introduction of fillers in the polymer matrix to prepare composite materials, such as polymer/nanofiller, polymer/molecular sieve, epoxy resin/silicate minerals, etc., can effectively inhibit the accumulation of space charge, improve the breakdown field strength [10,11,12,13,14,15], to avoid insulation breakdown. Therefore, it is of great significance to understand the effect of crosslinking additives and filler particles on the performance of power cable insulation specimens, which is important for the improvement in cable insulation strength and service life [16].

Lei’s team [17], through the study of a one-dimensional Al_2_O_3_ nano-air column insulation breakdown model, found that when the aperture size of the nano-air column is smaller than the average free range of the electrons along the parallel electric field or perpendicular to the direction of the electric field, the electron density in the nano-air gap is much lower than that required for the electron avalanche breakdown, which can limit the development of the electron impact ionization to a certain extent. At this time, the gas dielectric material is not following the traditional Townsend and Seitz theory of gas–solid discharge and avalanche breakdown [17,18,19], no matter how the strength of the applied electric field is increased, the nano-air gap will not produce insulation breakdown, the formation of insulation mode. Taking this as the theoretical basis, nano-fillers with a pore size structure are introduced into the crosslinked polyethylene matrix to improve the thermal conductivity of the composite material and, at the same time, to utilize the pore size structure of the fillers to inhibit the development of the electron impact ionization to realize the manipulation of avalanche breakdown and to improve the breakdown field strength of the composite material and the insulating strength of the crosslinked polyethylene insulating material. TS-1 [20] is a mesoporous ceramic material [21,22], belonging to the orthorhombic symmetric crystal system with an MFI-type molecular backbone structure, with a pore structure which is intersected by circular straight pores along the b-axis and elliptical sinusoidal pores along the a-axis. They have many unique and advantageous properties due to their nanoporous structure [23,24,25], which are widely used and concerned [26].

In this paper, the effects of crosslinking additive type and nanofiller TS-1 and its pore size structure on the breakdown voltage and thermal conductivity of crosslinked polyethylene composite insulation were investigated. Low-density polyethylene (LDPE) was selected as the matrix, and crosslinked polyethylene composites were prepared using dioctyl peroxide (DCP) and bis(tert-butyldioxyisopropyl) benzene (BIPB) as the crosslinking additives and nano-filler TS-1 as the filler, respectively, to utilize the synergistic effect of crosslinking additives and TS-1 to improve the thermal conductivity and insulation properties of crosslinked polyethylene insulation materials.

The microstructures of the samples were characterized using scanning electron microscopy (SEM) and transmission electron microscopy (TEM). The space charge distribution inside the different composites was also measured by the pulsed electroacoustic (PEA) method, and their dielectric constants, DC breakdown field strengths, resistivity, and thermal conductivity were tested. The effects of crosslinking additive types and nanofiller TS-1 and its pore size structure on the thermal conductivity and insulation properties of crosslinked polyethylene composites were analyzed.

## 2. Materials and Methods

### 2.1. Synthesis of XLPE

**Figure 1:**
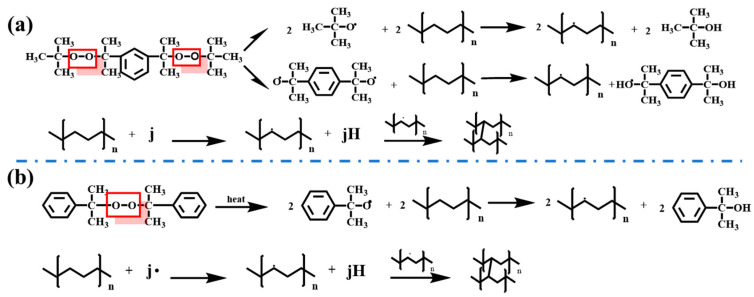
Reaction mechanism diagram of LDPE with crosslinking additives. (**a**) BIPB and LDPE; (**b**) DCP and LDPE.

XLPE was prepared by the melt blending and crosslinking method using low-density polyethylene LDPE as the matrix and DCP and BIPB as the crosslinking additives. Before the experiment, the raw materials were dried in a vacuum drying oven to remove the residual moisture. After the mixer reached the set temperature (120 °C), the low-density polyethylene was put into it, fully melted, and then added with crosslinking additives to mixing. The prepared products were placed in a vacuum drying oven and degassed at 70 °C to discharge the crosslinking by-products to obtain the final XLPE samples [19]. During the experiments, commercial low-density polyethylene was used as the base material to provide high-quality experimental samples with a stable performance and ensure the reproducibility of the experiments. XLPE/DCP is produced with DCP as the crosslinking additive, and XLPE/BIPB is obtained with BIPB as the crosslinking additive, which will be abbreviated as XLPE-1 and XLPE-2, respectively, in the following for simplicity and differentiation. The crosslinking of LDPE in the presence of two different crosslinking additives is shown in Figure 1.

Crosslinking additives thermally decompose at high temperatures to form free radicals, which react with the monomer molecular chain to form macromolecular chain radicals in the presence of the LDPE monomer to initiate the polymerization reaction. In the absence of a monomer, it will further decompose into primary radicals (where j represents various primary radicals) [27,28,29]. At the same time, the primary radicals will react with the surrounding polymer long chain, so that it loses free electrons to form long chain radicals due to the high activity of the primary radicals further reacting with other polymer long chains; the formation of the reaction chain continues to increase, polyethylene molecular chains crosslink with each other to ultimately form a three-dimensional mesh structure, and the high activity of free radicals is finally consumed in chain reaction termination [30,31,32,33].

**Figure 2:**
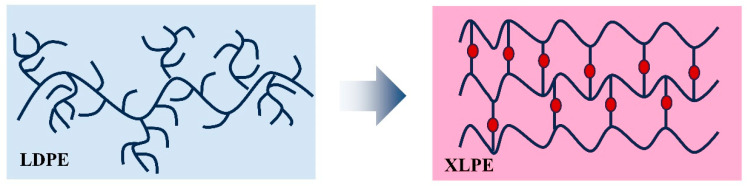
Schematic diagram of molecular structure changes during crosslinking process.

As shown in Figure 2, the crosslinking of LDPE using crosslinking additives converts the linear long chains of LDPE intertwined with each other into a three-dimensional mesh structure, and the crosslinking degree of the material increases. The increase in the degree of the crosslinking of the matrix and the formation of a three-dimensional network can increase the probability of the formation of thermal conductivity paths and a thermal conductivity network, so that the heat flow can be transferred along the filler, the thermal conductivity increases, the thermodynamic properties are improved [34], and the structure of the material will also affect the insulating properties of the material; with the increase in the degree of crosslinking and formation of the three-dimensional network, the material’s internal free volume decreases, the movement of polymer molecular chains are restricted, and electrical properties, such as volume resistivity, breakdown field strength, and others [35], will also be improved to a certain extent.

### 2.2. Synthesis of TS-1

The nanofiller titanium–silicon molecular sieve TS-1 was prepared by in situ hydrothermal synthesis using Ti_2_(SO)_4_ as the titanium source, ethyl orthosilicate (TEOS) as the silicon source, and tetra propylammonium hydroxide (TPAOH) as the templating agent. We weighed 55 g of ultrapure water and 48.8 g of tetra propylammonium hydroxide in a beaker, mixed thoroughly and heated slowly to 80 °C, added 16 g of ethyl orthosilicate dropwise during the period, and kept the reaction with mechanical stirring for 1 h. After stopping the heating and waiting for the mixture to be cooled down to 0 °C, the formulated titanium source solution (1.8 g of titanium sulphate dissolved in 3.5 g of H_2_O_2_ and 5 mL of H_2_O) was added dropwise into the above silica sol and then hydrolysed in an ice bath for 20 min and slowly warmed up to 80 °C and reacted at this constant temperature for 3 h. At the end of the reaction, the mixture was transferred to a high-pressure reactor and hydrothermally reacted at 120 °C for 24 h. The precipitates were separated by centrifugation. The precipitate was separated by centrifugation and washed several times to remove the excess alkaline material. Finally, the separated precipitate was vacuum-dried at 80 °C for 24 h to remove the residual water to obtain white powder TS-1 [18].

### 2.3. Preparation of TS-1/XLPE Nanocomposites

TS-1/XLPE nanocomposite films were prepared by a combination of melt blending and crosslinking hot pressing. The TS-1/XLPE-1 and TS-1/XLPE-2 nanocomposites with filler contents of 0.5, 1.0, 1.5, and 2.0 wt.% were prepared by melt blending two matrices (XLPE-1 and XLPE-2) with TS-1 nanofillers using a torque rheometer (RM-200C). The bulk solid composites obtained after melt blending were sheared into small pieces and thermally compression molded using a flat vulcanizing plate to obtain the corresponding composite films [18,19,36].

In order to easily differentiate between these samples, we prepared the XLPE-1-based samples, labeled as 1–5#. The XLPE-2-based samples are labeled as 6–10#.

## 3. Results and Discussion

### 3.1. TEM

**Figure 3:**
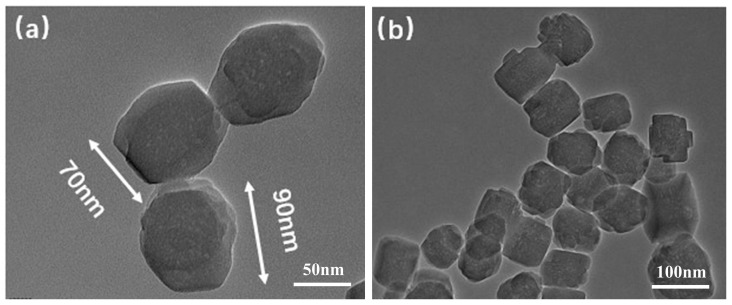
TEM images of TS-1 at different magnifications. (**a**) TEM image at a scale of 50 nm, (**b**) TEM image at a scale of 100 nm.

The morphology and structure of the experimentally synthesized TS-1 were characterized using transmission electron microscopy. The samples were pretreated before the experiment, ultrasonically dispersed so that they were fully dispersed in anhydrous ethanol solution, and a small amount of the samples were placed on a copper grid for observation and analysis, and the results of the experiment are shown in Figure 3. It can be seen from the figure that the TS-1 nanoparticles are cubic in shape, with a uniform particle size between 70 and 100 nm, good dispersion, and no obvious agglomeration and stacking phenomenon. The pore size structure of TS-1 nanoparticles can be clearly observed under large magnification.

### 3.2. SEM

**Figure 4:**
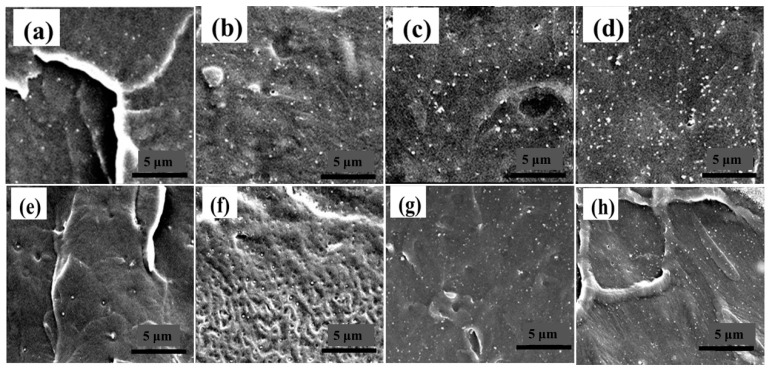
Cross-section SEM images of TS-1/XLPE-1 nanocomposites. (**a**) The 0.5 wt.% TS-1/XLPE-1 nanocomposite; (**b**) 1.0 wt.% TS-1/XLPE-1 nanocomposite; (**c**) 1.5 wt.% TS-1/XLPE-1 nanocomposite; (**d**) 2.0 wt.% TS-1/XLPE-1 nanocomposite; (**e**) 0.5 wt.% TS-1/XLPE-2 nanocomposites; (**f**) 1.0 wt.% TS-1/XLPE-2 nanocomposites; (**g**) 1.5 wt.% TS-1/XLPE-2 nanocomposites; (**h**) 2.0 wt.% TS-1/XLPE-2 nanocomposites.

The uniformity of TS-1 nanofiller dispersion in the XLPE matrix affects the insulation strength and thermomechanical properties of composites. In order to investigate the dispersion of the nanofiller TS-1 and to analyze the effect of the uniformity of filler dispersion on the energy storage properties of the composites, the cross-sections of TS-1/XLPE composite films were characterized by field emission scanning electron microscopy (SEM). To avoid the influence of impurities, dust, and moisture, the surface of the samples was cleaned with anhydrous ethanol solution, and the samples were placed in a vacuum drying oven for drying treatment, quenched in liquid nitrogen, sprayed with gold, and cross-sectioned for SEM characterization, and the results of the experiments are shown in Figure 4. Figure 4a–d show the SEM images of the quenched cross-section of the TS-1/XLPE-1 nanocomposites (samples #2#–5#), which corresponded to the DCP-based doping levels of TS-1 as the crosslinking additives were 0.5, 1.0, 1.5, and 2.0 wt.%, respectively, and Figure 4d–g correspond to the SEM images of the quenched sections of samples #7#–10#, which were crosslinked with BIPB as the crosslinking additive.

The black and gray parts are XLPE-1 and XLPE-2 matrices, and the relatively bright parts are filler particles TS-1. Combined with the experimental images, it can be seen that the area of the bright parts gradually increases with the increase in the TS-1 content. When the filler content is low, the TS-1 nanoparticles are well dispersed and uniformly distributed in the XLPE matrix. With the increase in filler, large-size agglomerated filler particle clusters appeared because the polarity difference between the filler and the matrix was large, and with the increase in filler content, the filler with larger polarity preferentially agglomerated. The agglomeration of TS-1 would make it unable to combine with the three-dimensional mesh structure of the XLPE efficiently, and the compatibility between the filler and matrix would be reduced, and voids and defects appear inside the material. As shown in Figure 4c,d,g,h, with the increase in the TS-1 content in the cross-section, SEM images can be observed in the voids to defects; these voids and defects are due to the particles, and the matrix in the binding force is small, the compatibility is poor, and pores and defects form in the filler–matrix interface [37].

### 3.3. Thermal Conductivity Analysis (TC)

The intrinsic thermal conductivity of the matrix as well as the type of filler, particle size, and state of dispersion in the matrix directly affect the thermal conductivity (TC) values of the composites [38,39,40,41]. In this study, in order to investigate the effect of crosslinking additives and filler particles TS-1 on the thermal conductivity of crosslinked polyethylene, the thermal conductivity of TS-1/XLPE composites was measured using the laser flash vaporization method. Before the experiment, the samples were pretreated and cut into standard sizes with a diameter of 25.4 mm and a thickness of 1 mm, and the graphite was uniformly sprayed on the surface of the sample pieces to ensure that the light energy absorbance and infrared emissivity were the same at different test sites and to improve the accuracy of the counting test.

**Figure 5:**
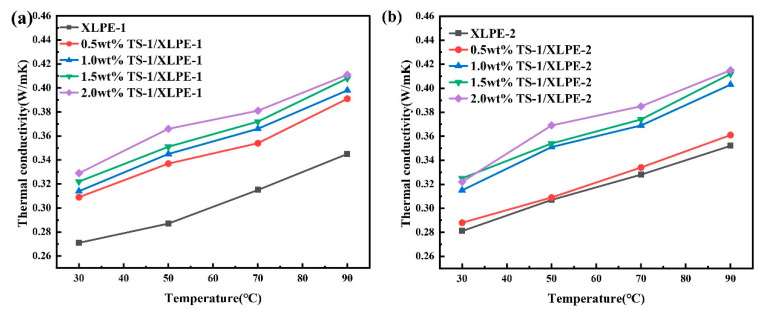
Thermal conductivity TS-1/XLPE-1 and TS-1/XLPE-2 nanocomposites. (**a**) TS-1/XLPE-1 nanocomposites; (**b**) TS-1/XLPE-2 nanocomposites.

The thermal conductivity versus temperature curves of TS-1/XLPE nanocomposites containing different mass fractions of TS-1 nanofillers are shown in Figure 5. Crosslinked polyethylene nanocomposites are the key components of the insulation layer of power transmission cables, and their working temperature is not much different from the outside temperature, so generally not higher than 50 °C. However, with the prolongation of the working time, the internal heat of the cable cannot be dissipated in time, the temperature of the insulation layer will also rise, and in order to simulate the crosslinked polyethylene insulation materials in extreme conditions, the experimental testing process test temperature range is set to 30–90 °C.

From Figure 5, it can be seen that the TC of XLPE matrix and TS-1/XLPE nanocomposites are positively correlated with temperature, and the thermal conductivity increases gradually with increasing temperature. This is because high temperatures increase the vibration of atoms and molecules, which increases the rate of thermal conduction and improves the thermodynamic properties of the materials. The TC values of the pure crosslinked polyethylene matrix XLPE-1 and XLPE-2 are 0.271 and 0.281 W/mK, respectively, at 30 °C. The thermal conductivity of LDPE is measured to be 0.204 W/mK under the same experimental conditions, and the thermomechanical properties of the materials have been improved by the addition of crosslinking additives. The TC values of TS-1/XLPE composites with the introduction of nanofiller TS-1 were higher than those of the uncompounded XLPE matrix. When the content of TS-1 reaches 2.0 wt.%, the TC of the TS-1/XLPE-1 composites is 0.329, and that of TS-1/XLPE-2 is 0.332 W/mK, which is 21.4% and 18.2% higher than that of the XLPE matrix. Based on the experimental test results, it can be seen that the thermal conductivity of the composite with the XLPE-2 matrix is higher than that of the system with the XLPE-1 matrix. This result indicates that the use of BIPB as a crosslinking additive is more favorable for the improvement in the thermo-mechanical properties of the crosslinked polyethylene composites.

**Figure 6:**
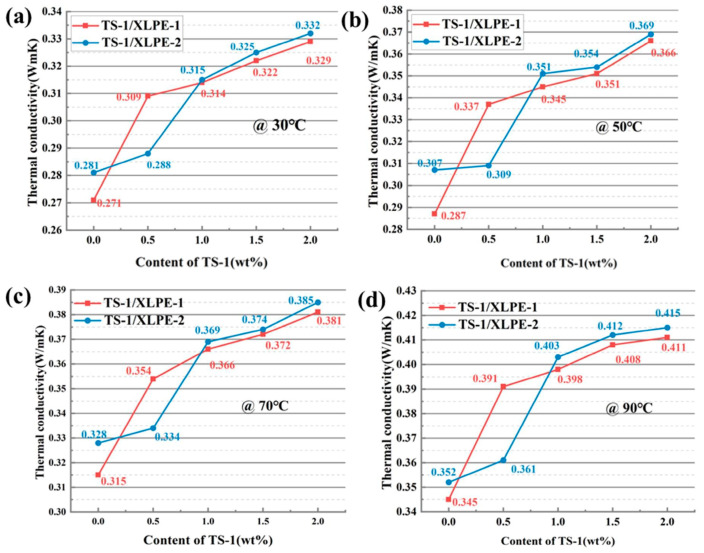
Line graph of thermal conductivity of TS-1/XLPE nanocomposites with filler content at different temperatures. (**a**) Thermal conductivity trend at 30 °C, (**b**) thermal conductivity trend at 50 °C, (**c**) thermal conductivity trend at 70 °C, (**d**) thermal conductivity trend at 90 °C.

Figure 6 shows the variation curves of thermal conductivity with TS-1 content for two TS-1/XLPE nanocomposites at different temperatures. Different matrices have different thermal conductivities at the same temperature and filler mass fraction, which further illustrates that the type of crosslinking additive affects the thermal properties of the composites. According to Figure 6, it can be seen that the thermal conductivity of TS-1/XLPE nanocomposites increases with the increase in the TS-1 content, which is due to the fact that the nanoparticles TS-1 will interact with the three-dimensional mesh structure of the XLPE, increasing the probability of the formation of thermal conductive pathways and thermal conductive networks, so that the heat flow can be transferred along the filler, the thermal conductivity increases, and the thermal properties of the composites are improved. However, the intrinsic thermal conductivity of TS-1 is low, and the introduction of nanofillers will reduce the free volume inside the material, and the movement of the polymer molecular chains is restricted, so the thermal conductivity enhancement of TS-1/XLPE composites is limited.

### 3.4. Breakdown Strength (E_b_)

**Figure 7:**
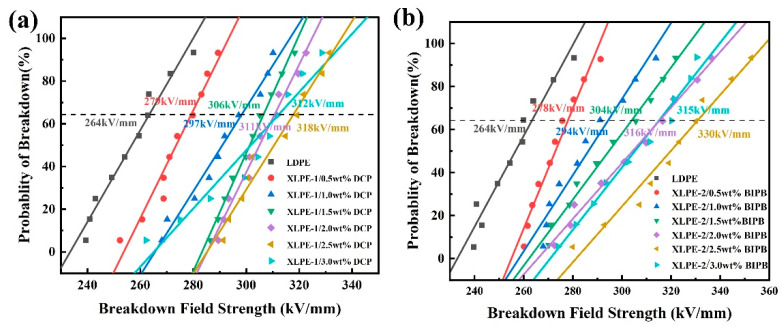
Breakdown field diagrams of crosslinked polyethylene and LDPE prepared by adding different crosslinking additives. (**a**) Breakdown field strength of DCP/LDPE; (**b**) BIPB/LDPEDCP/LDPE.

Breakdown performance (E_b_) was measured using a breakdown tester at room temperature at a ramp rate of 500 V/s. The test was performed in a vacuum environment to remove water molecules from the sample. Prior to testing, the samples were first pretreated, surface-cleaned to remove surface stains, and dried in a vacuum environment to eliminate the effect of water molecules in the samples on the reliability of the data. The E_b_ of LDPE and the two matrices XLPE-1 and XLPE-2 were comparatively analyzed to investigate the effect of crosslinking additives and their types on the breakdown properties of LDPE, and the experimental results are shown in Figure 7. From the figure, it can be seen that the breakdown properties of the crosslinked polyethylene matrix produced by different types of crosslinking additives are somewhat different. When the doping level of the crosslinking additive is 2.5 wt.%, XLPE-1 and XLPE-2 have the best breakdown properties, with maximum values of E_b_ of 318 kV/mm and 330 kV/mm, respectively, which are increased by 19.25% and 25.66%, respectively, compared with LDPE (264 kV/mm). The increase in the E_b_ value of the material with the addition of crosslinking additives is due to the fact that the addition of DCP and BIPB makes the independent molecular chains in LDPE interconnected to form a three-dimensional network, which makes it difficult to penetrate the specimen when the voltage is applied and improves the breakdown field strength of the crosslinked polyethylene composite material.

**Figure 8:**
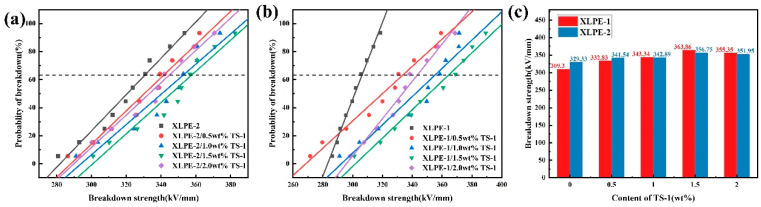
Breakdown strength of TS-1/XLPE nanocomposites with different TS-1 concentrations, (**a**) TS-1/XLPE-1 nanocomposites, (**b**) XLPE-2 nanocomposites, (**c**) comparative histogram of breakdown field strength.

The characteristic curve of TS-1/XLPE composites with the TS-1 content is shown in Figure 8. From the figure, it can be seen that E_b_ increases and then decreases with the increase in the TS-1 nanofiller content. The E_b_ of TS-1/XLPE-1 nanocomposite is significantly higher than other ratios at 1.5 wt.% filler doping, which is 363 kV/mm, and the maximal E_b_ of the TS-1/XLPE-2 nanocomposite is 356 kV/mm. In conjunction with Figure 8c, it can be seen that both the type of crosslinking additive and filler content affect the DC breakdown field strength of crosslinked polyethylene nanocomposites. When the nanofiller content is low, the Eb of the composites with the type of crosslinking aid is more affected. With the increase in the TS-1 content, the effect of crosslinking additive type on the Eb of the material diminishes, and the E difference between XLPE-1 and XLPE-2 gradually decreases. This further illustrates the synergistic effect of nanofillers and crosslinking additives on the breakdown properties of crosslinked polyethylene composites.

**Figure 9:**
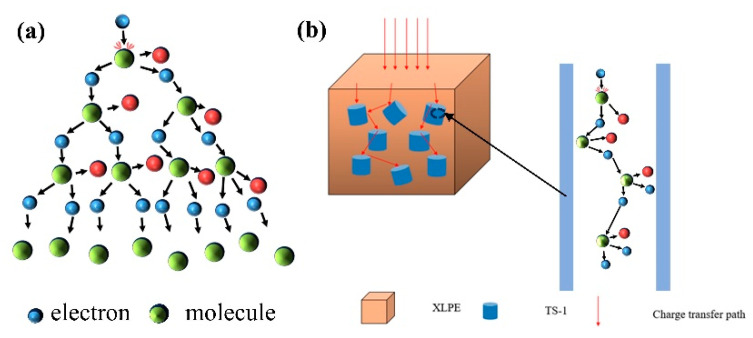
Schematic diagram of the mechanism of enhancement of breakdown field strength. (**a**) Schematic diagram of electron impact ionization conduction; (**b**) schematic diagram of the blocking effect of TS-1 on charge injection and transfer in XLPE matrix.

The enhancement of the breakdown field strength of TS-1/XLPE is a result of the combination of several factors. On the one hand, due to the TS-1 nanoparticles showing intrinsically excellent electrical insulation performance, higher than the crosslinked polyethylene body, the crosslinked polyethylene and TS-1 composite material insulation performance is improved, and the maximum applied electric field strength is improved; on the other hand, the introduction of the TS-1 will be after the rearrangement of the material internal space charge, where the electric field distribution is changed, the free volume is reduced, the electric field is homogenized, and the filler particles will impede the charge conduction; the specific principle is shown in Figure 9. In addition, the pore size structure of TS-1 can inhibit the impact ionization of electrons, block the formation of electron avalanche breakdown, and truncate the conduction of electric dendrites in order to improve the breakdown field strength of polypropylene composites. However, when the mass fraction of TS-1 nanoparticles increases to a certain value, the high filler content causes the nanoparticles to overlap, creating voids and cavities, forming a breakdown weak point, which leads to a decrease in the DC breakdown field strength of the nanocomposites. 

### 3.5. Resistivity (R_v_)

The resistivity (R_v_) of the composite material reflects the impedance of the material to the current and is an important performance parameter of the insulating material. In this experiment, a three-electrode test system was used to measure the volume resistance of samples 1–10#. And in order to ensure the credibility of the experimental data and avoid the data error caused by the operation, the average value of each sample is taken as the R_v_ of material after repeating the measurement several times.

**Figure 10:**
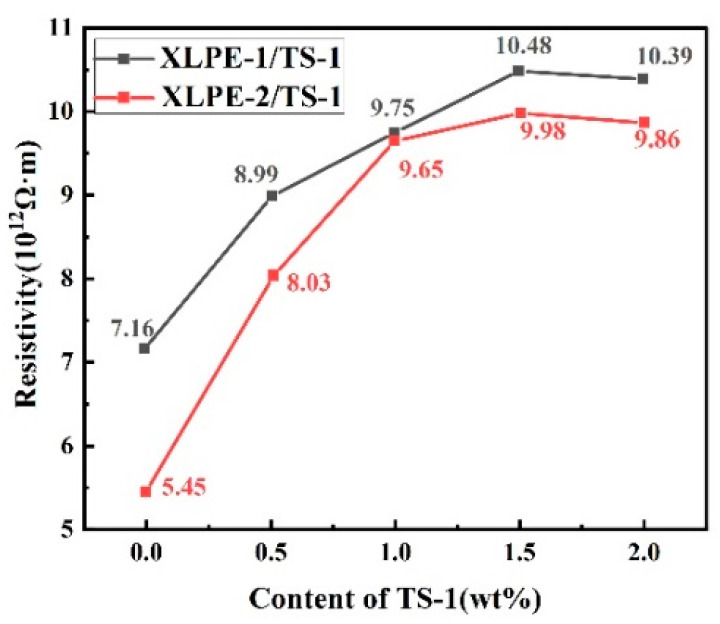
Volume resistivity of TS-1/XLPE nanocomposites with different TS-1 content.

The test results are shown in Figure 10. The resistivity of samples 1–5# with DCP as the crosslinking agent is generally higher than that of samples 6–10# (TS-1/XLPE nanocomposites) with BIPB as the crosslinking agent at the same addition amount. The DC resistivity of the same set of samples first increased and then decreased with the increase in the TS-1 nanoparticle content. When the content of TS-1 was 1.5 wt.%, the R_v_ had maximum values of 10 × 10^12^ Ω·m and 9 × 10^12^ Ω·m, respectively. The results showed that the crosslinker and TS-1 nanoparticles acted together to affect the R_v_ of the TS-1/XLPE in the matrix The addition of TS-1 nanoparticles can effectively improve the R_v_ of the material. The resistivity test results corresponded to the DC breakdown test results, and the TS-1/XLPE nanocomposites had the highest E_b_ and R_v_ when the TS-1 content was 1.5 wt.%.

### 3.6. Dielectric Constant $\left(E_{r}\right)$

**Figure 11:**
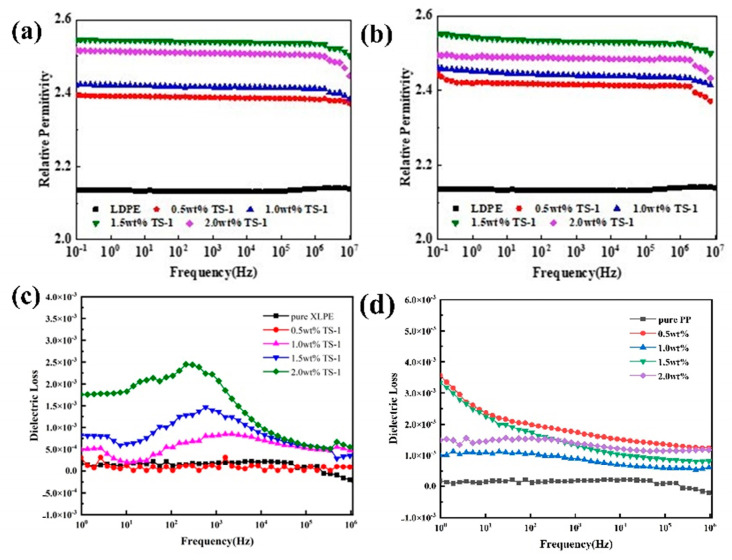
Dielectric constant with frequency curves of XLPE/TS-1 nanocomposites. (**a**) XLPE-1/TS-1 nanocomposites; (**b**) XLPE-2/TS-1 nanocomposites; curves of dielectric loss versus frequency for XLPE/TS-1 nanocomposites. (**c**) XLPE-1/TS-1 nanocomposite; (**d**) XLPE-2/TS-1 nanocomposite.

The dielectric constants of the samples were tested using broadband dielectric spectroscopy from 10^−1^ to 10^6^ Hz, and the experimental results are shown in Figure 11. The introduction of high dielectric nanofiller TS-1 can increase the dielectric constant of the crosslinked polyethylene composites compared to the pure matrix to obtain high dielectric crosslinked polyethylene-based composites. In the frequency range of 10^−1^–10^6^ Hz, the $E_{r}$ of the samples was basically stable without big changes, and the dielectric constant increased and then decreased with the increase in TS-1 doping, but they were all higher than that of the pure XLPE. When the concentration of TS-1 nanoparticles was 1.5 wt.%, the $E_{r}$ had maximum values of 2.53 and 2.54, respectively. TS-1 is a polar filler and will undergo steering polarization under the action of an applied electric field. At the same time, due to the large specific surface area of TS-1, it is uniformly dispersed in the XLPE matrix through interaction with the matrix, forming a good nanoparticle–polymer matrix interface and introducing interfacial polarization. The $E_{r}$ of TS-1/XLPE nanocomposites increases significantly under the combined effect of the two polarization effects. When the nanoparticle content is too large, nanoparticle aggregation restricts the movement of molecular chains, leading to a decrease in the planned performance and a decrease in the relative dielectric constant.

The dielectric constant of a composite material is related to the polarization behavior of the electric dipole inside the material. Under the action of an applied electric field, the macroscopic dipole moment inside the dielectric material moves along the direction of the electric field to produce polarization, but the establishment of the polarization process needs some time. When the frequency is low, the polarization process can keep up with the electric field, and the dielectric constant is high. As the frequency increases too high, the carriers do not have enough time to follow and react to the change in the external electric field, resulting in a decrease in the relative dielectric constant. The dielectric constant of TS-1/XLPE nanocomposites decreases at a frequency of 10^6^ Hz. Meanwhile, according to the experimental images, it can be seen that the dielectric constants of different composites with a crosslinked polyethylene matrix are amateurishly different, which further indicates that the type of crosslinking additives also affects the dielectric properties of the composites.

### 3.7. Space Charge Analysis

**Figure 12:**
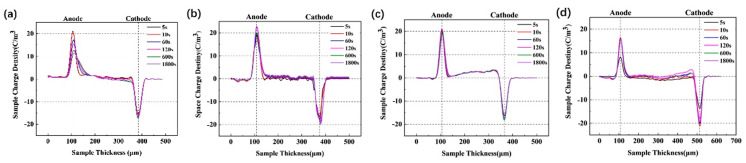
The space charge density of XLPE and TS-1/XLPE nanocomposites: (**a**) XLPE-1, (**b**) XLPE-2, (**c**) XLPE-1/1.5 wt.% TS-1, (**d**) XLPE-2/1.5 wt.% TS-1.

The distribution of the space charge of the samples was measured using the electroacoustic pulse method (HEYI-PEA-PT1). The applied electric field strength was 20 kV/mm at room temperature. The thickness of the insulating layer is plotted as the horizontal coordinate, and the density of the space charge is plotted as the vertical coordinate, and the result is shown in Figure 12.

After an applied voltage is applied to the upper surface of the sample, negatively charged free electrons are injected from the cathode, while positively charged holes enter the material from the anode [5,42]. The charge builds up rapidly near the cathode of the XLPE, and the space charge at the electrode increases with time and has a limiting value at 120 s. The space charge of the specimens with two different matrices shows the same pattern. By comparing Figure 12a,b, the effect of the type of crosslinker on the space charge content inside the crosslinked polyethylene matrix is evident. The TS-1/XLPE nanocomposites were prepared with the two different crosslinked polyethylene matrices mentioned above, respectively, and the space charge densities near the anode and cathode of the composites were much lower compared to that of pure XLPE. This is because the introduction of TS-1 nanoparticles introduces deep traps inside the material, which can trap free electrons and inhibit the migration of carriers [17,43], thus avoiding the accumulation of space charge, which results in the distortion of the electric field, improving the insulating strength of the crosslinked polyethylene-based composites.

## 4. Conclusions

In order to improve the insulation lightness and thermal conductivity of crosslinked polyethylene composites, a TS-1/XLPE binary composite nanomodified system was constructed, and the effects of the type of crosslinking additives (DCP and BIPB) as well as TS-1 nanofillers on the properties of crosslinked polyethylene-based nanocomposites in terms of thermal conductivity, dielectric constant, breakdown field strength, volume resistivity, and space charge distribution were investigated. It was found experimentally that the type of crosslinking agent as well as the filler content affect the properties of the composites. Under the action of crosslinking additives DCP and BIPB, LDPE independent molecular chains are connected to each other to form a three-dimensional network, which improves the probability of the formation of thermal conductivity paths and a thermal conductivity network, so that the thermal conductivity of the material increases, and the TC values of the purely crosslinked polyethylene matrices, XLPE-1 and XLPE-2, are elevated to 0.271 and 0.281 W/mK. With the addition of an applied electric field, the complex three-dimensional molecular mesh structure makes it difficult for the specimen to be penetrated, so that the breakdown field strength is increased. The optimum doping level of 2.5 wt.% of the crosslinking additives resulted in the best breakdown performance of the crosslinked polyethylene matrix (XLPE-1 and XLPE-2), reaching 318 kV/mm and 330 kV/mm, respectively, which is an increase of 19.25% and 25.66%, respectively, in comparison with the low-density polyethylene. The effect of crosslinker type on the sample performance decreases with higher TS-1 doping. Due to its excellent electrical insulation properties, TS-1 can introduce deep traps inside the material to capture free electrons and inhibit carrier migration, avoiding space charge accumulation and electric field distortion. The unique nanopore structure of TS-1 inhibits electron impact ionization and improves the breakdown field strength and insulation strength of crosslinked polyethylene matrix composites. In addition, due to the good compatibility of TS-1 nanoparticles with the crosslinked polyethylene matrix, they can be uniformly dispersed in the matrix to form a good filler–matrix interface, and the dielectric properties of the composites can be improved under the joint action of dipole steering polarization and interface polarization. When the TS-1 concentration is 1.5 wt.%, the TS-1/XLPE-1 and TS-1/XLPE-2 nanocomposites have the optimal performance, with breakdown field strengths of 363 and 356 kV/mm and resistivities of 10 × 10^12^ and 9 × 10^12^ Ω·m, respectively. The relative permittivity was 2.53 and 2.54, and the thermal conductivity was 0.415 and 0.411. The introduction of crosslinking additives and TS-1 nanoparticles improved the insulating properties and thermal stability of the crosslinked polyethylene nanocomposites, which is of great significance in improving the life and performance of the materials for high-voltage direct current transmission cables.

## Data Availability

Data are contained within the article.
